# The biological effects of microencapsulated organic acids and botanicals induces tissue-specific and dose-dependent changes to the *Gallus gallus* microbiota

**DOI:** 10.1186/s12866-020-02001-4

**Published:** 2020-11-02

**Authors:** Kristina M. Feye, Christina L. Swaggerty, Michael H. Kogut, Steven C. Ricke, Andrea Piva, Ester Grilli

**Affiliations:** 1 U.S. Department of Agriculture, Agricultural Research Service, Southern Plains Agricultural Research Service, 2881 F and B Road, College Station, TX 77845 USA; 2 Meat Science & Animal Biologics Discovery Program, Department of Animal and Dairy Sciences, University of Wisconsin, Madison, WI USA; 3 DIMEVET, University of Bologna, Ozzano Emilia, Bologna, Italy; 4 Vetagro S.p.A, Reggio Emilia, Italy; 5 Vetagro Inc., Chicago, IL USA

**Keywords:** Botanicals, Ileum, Jejunum, Microbiota, Microencapsulated, Organic acids

## Abstract

**Background:**

Microencapsulated organic acids and botanicals have the potential to develop into important tools for the poultry industry. A blend of organic acids and botanicals (AviPlus®P) has previously shown to reduce *Salmonella* and *Campylobacter* in chickens; however, changes to the microbiota of the jejunum and ileum have not been evaluated. Microbiota diversity is linked to, but not correlated with, the efficacy of natural products; therefore, understanding the effects on the microbiota is necessary for evaluating their potential as an antibiotic alternative.

**Results:**

Ileal and jejunal segments from control and supplement-fed chickens (300 and 500 g/metric ton [MT]) were subjected to alpha diversity analysis including Shannon’s diversity and Pielou’s Evenness. In both analytics, the diversity in the ileum was significantly decreased compared to the jejunum irrespective of treatment. Similarly, beta diversity metrics including Bray-Curtis dissimilarity index and Weighted Unifrac Distance Matrix, were significant (Q < 0.05) for both tissue and treatments comparisons. Alpha and beta diversity analytics indicated compartmentalization effects between the ileum and jejunum. Additionally, analysis of communities in the microbiota (ANCOM) analysis showed *Lactobacilliaceae* predominated the total operational taxonomic units (OTU), with a stepwise increase from 53% in the no treatment control (NTC) to 56% in the 300 g/MT and 67% in the 500 g/MT group. *Staphylococcaceae* were 2% in NTC and 2 and 0% in 300 and 500 g/MT groups. *Enterobacteriaceae* decreased in the 500 g/MT (31%) and increased in the 300 g/MT (37%) compared to the NTC (35%). *Aerococcaceae* was 0% for both doses and 7% in NTC. *Ruminococcaceae* were 0% in NTC and 2 and 1% in the 300 and 500 g/MT. These changes in the microbial consortia were statistically (*Q* < 0.05) associated with treatment groups in the jejunum that were not observed in the ileum. Least discriminant analysis effect size (LEfSE) indicated different changes directly corresponding to treatment. *Enterobacteriacea*e demonstrated a stepwise decrease (from NTC onward) while *Clostridiaceae*, were significantly increased in the 500 g/MT compared to NTC and 300 g/MT (*P* < 0.05).

**Conclusion:**

The bioactive site for the microencapsulated blend of organic acids and botanicals was the jejunum, and dietary inclusion enhanced the GIT microbiota and may be a viable antibiotic alternative for the poultry industry.

## Background

The public concerns associated with the use of antibiotics in poultry production, and animal agriculture in general, necessitates research into acceptable natural alternatives that promote feed efficiency and food animal health while reducing the burden of foodborne disease. Stepping back from the refined pharmacological fungal metabolites traditionally used in animal agriculture, plant secondary metabolites and essential oils are an attractive avenue of development for use by the poultry industry [1]. Research indicates bioactive natural compounds can decrease the microbial burden on the immune system and promote feed efficiency by improving digestibility and gastrointestinal (GIT) morphology [2–4] and intestinal mucosal barrier function [5] in poultry. Additionally, essential oils and other natural compounds are generally regarded as safe and can be multi-modal in their activation effects including antimicrobial, anti-parasitic, therapeutic, anti-inflammatory, and chemotherapeutic properties [1, 6, 7]. There are numerous recent reviews highlighting natural compounds for their potential to serve as antibiotic alternatives including, but not limited to, cinnamon [8], oregano [9], organic acids [10, 11] and others [12].

The GIT microbiota actively participates in homeostatic function, nutrient digestion, and biotransformation of compounds. The symbiotic relationship between host commensal microorganisms and the immune system facilitate immune tolerance and development and can have peripheral consequences to overall health and food animal feed efficiency [13–16]. Additionally, as the microbiota directly interacts with feed matrices, natural compounds must not adversely impact the microbiota community structure and stability. Sufficient evidentiary support must therefore demonstrate that the microbiota does not render the natural compounds inert nor that the biotransformation results in bactericidal effects that reduce diversity that corresponds with decreased absorption of nutrients and compounds [17–19].

Compartmentalization, or localization to a particular section the GIT, is important, though often overlooked in poultry feed amendment studies [20]. The activity of natural compounds should result in changes to the compartment of activity which would provide knowledge related to the changes within the microbial community structure and may ultimately provide insight into the biology driving these effects. As microencapsulation technology continues to evolve, the targeted delivery of natural compounds through the harsh environment of the crop to their intended location further down the GIT may serve to improve biological activity [21]. Additionally, a study by Grilli and colleagues showed that microencapsulation allows for the slow release of organic acids in the small intestine of broilers [22].

There are numerous poultry-specific studies in the literature that evaluate the role of essential oils and other natural products. For example, thymol has been shown to be anti-inflammatory with the ability to modulate the microbiota [23], reduce the effects necrotic enteritis [24], and vanillin exhibits antibiotic-like effects [25, 26]. Organic acids also show promise as feed amendments in poultry [11]. Dietary supplementation with benzoic acid influences gut microbial populations [27], and the addition of organic acids and essential oils improves performance and increases disease resistance [10], while propionic and formic acid supplementation improves carcass traits [28].

Clearly, individual feed additive components have been studied; however, the combinatorial effects of the microencapsulated blend of organic acids and botanicals used in the current study remains to be understood. In a previous study by Mohammadi Gheisar et al., broilers fed with microencapsulated organic acids and botanicals had improved performance and feed efficiency as well as an increase in *Lactobacillus* counts in the feces [29]. As changes in the intestinal microbiota can be one of the drivers to growth performance, one of the objectives of this study was to investigate the impact of microencapsulated organic acid and botanicals on the microbiota. Moreover, our laboratory recently performed a kinome analysis of ileal and jejunal segments collected from broilers on the microencapsulated diet and showed key differences in immune and metabolic signaling pathways compared to controls indicating tissue-specific differences that are directly attributed to the amended diet [30]. Therefore, the other objective of the present study was to evaluate the potential compartmentalized effects of the microencapsulated blend of organic acids and botanicals on the ileum and jejunum populations. It is important to conduct feed additive studies in vivo; therefore, the commercial broiler by-product chickens used in this study were selected as they are representative birds used in today’s poultry production. By evaluating community structure and composition, it will be possible to determine if there are any effects on the microbiota due to bioactivity of organic acids and botanicals in specific compartments of the GIT.

## Results

### Animal health, well-being, and chick weights

Chickens were monitored daily and no mortality, behavioral changes, or other animal welfare concerns were observed during the course of the study for the controls or those on the supplemented diets. Other than the dietary supplement that included the microencapsulated blend of organic acids and botanicals, the chicks were not administered any medications or other therapeutic interventions during the study. At placement, there were no differences (*P* > 0.05) in chick weight between the three groups for either experimental replicate (no treatment control [NTC] = 0.044 ± 0.001 kg; 300 g/MT = 0.045 ± 0.0003 kg; 500 g/MT = 0.044 ± 0.001 kg). Group weights were determined at the conclusion of each study, and supplement-fed chicks were slightly heavier (300 g/MT = 0.525 ± 0.007 kg; 500 g/MT = 0.526 ± 0.002 kg) than the NTC chicks (0.522 ± 0.003 kg); however, these differences were not significant (*P* > 0.05).

### Alpha diversity analysis

**Fig. 1:**
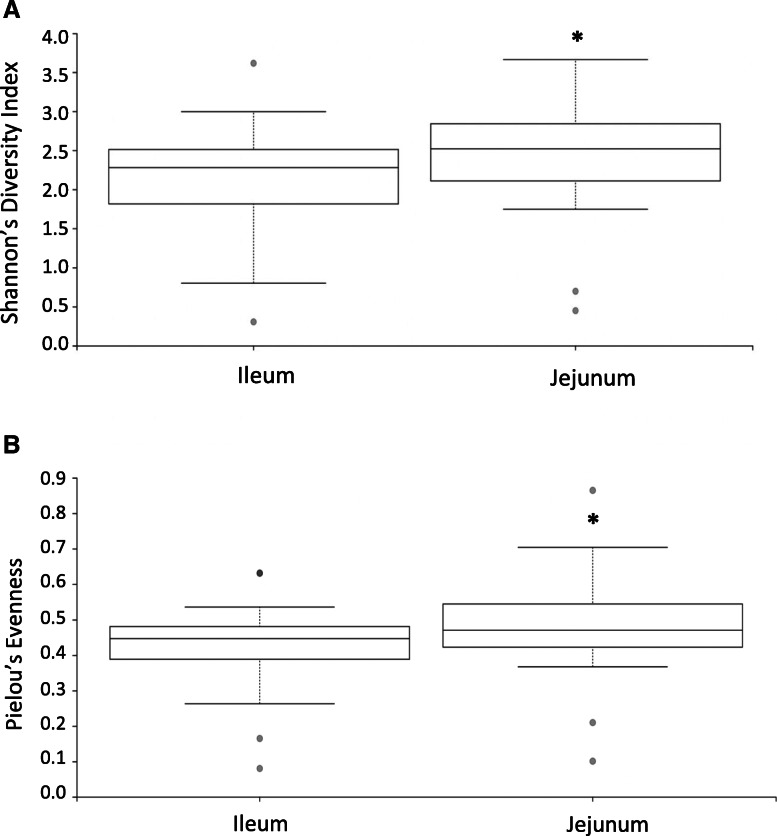
Alpha diversity matrix. **a** Shannon diversity index of gut compartment. **b** Pielou’s evenness by gut compartment. The asterisk represents a significant difference between the ileum and jejunum (Q < 0.05; main effect *P* < 0.05). Evidence indicates compartmentalization was maintained

For each independent experiment (*n* = 2), five ileal and jejunal samples were collected. In total, samples from 10 chickens were included in the bioinformatics analyses per treatment for all analytics. Evenness and richness are two essential components to alpha diversity. Therefore, taken together, both metrics are able to assess changes in alpha diversity due to location or treatment. The effects of location were significant (*P* < 0.05) for Shannon’s diversity index (Fig. 1a) and Pielou’s evenness (Fig. 1b) comparing the ileum and jejunum. There was a significant (*P* < 0.05) decrease in Shannon’s diversity index and Pielou’s evenness metric for the ileum. Meaning, species richness and the even distribution of that richness across the ileum is less than that of the jejunum.

### Beta diversity analysis

**Fig. 2:**
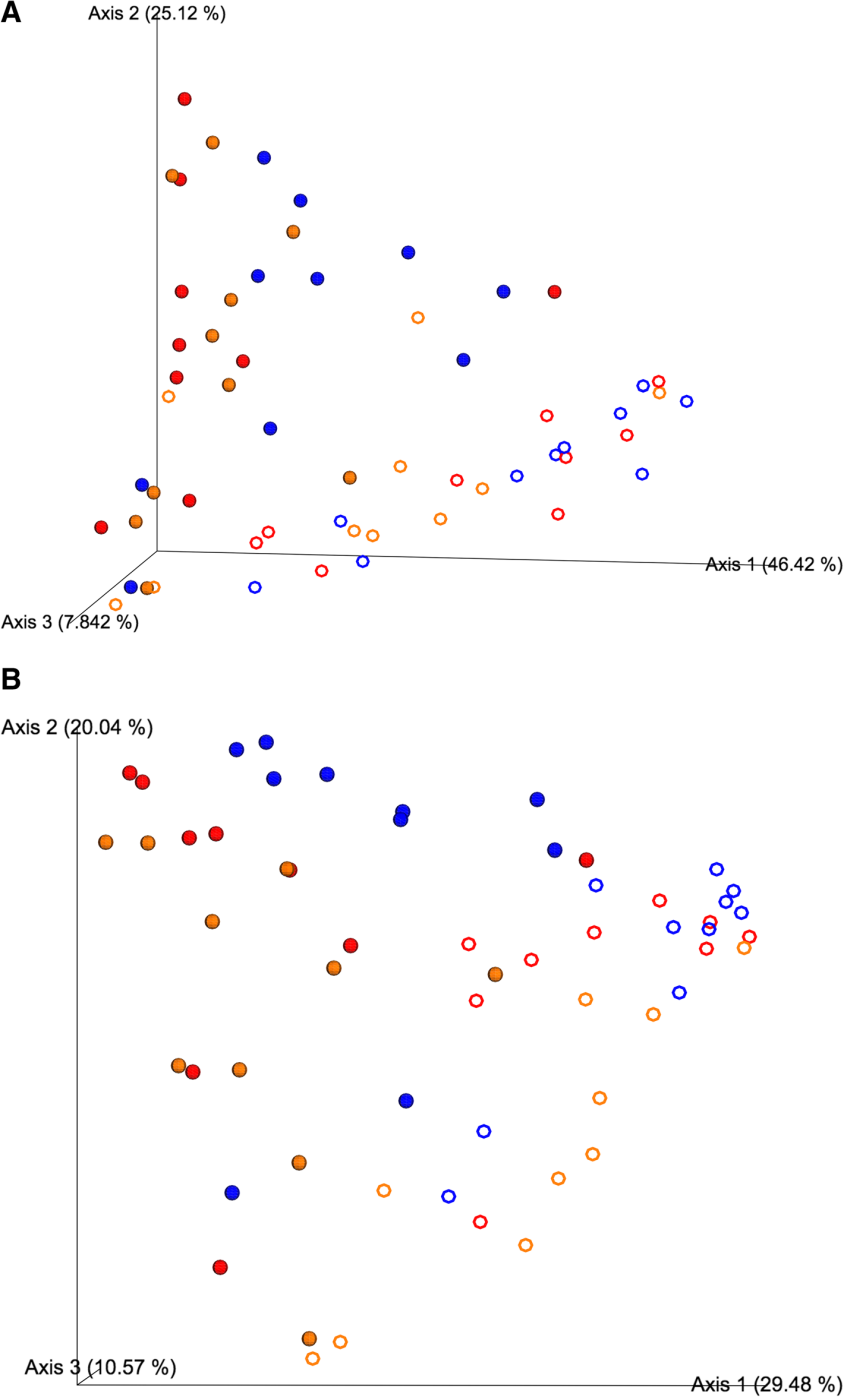
Beta diversity matrix. **a** Weighted unifrac distance matrix. **b** Bray-Curtis dissimilarity index. Shape coding: Sphear: Ileum; Ring: jejunum. Color: Red: NTC; Blue: 300 g/MT; Gold: 500 g/MT. Significant differences exist for compartmentalization. The effect of treatment was demonstrated throughout the study

For each study (*n* = 2), a total of 5 samples were collected. In total, samples from 10 chickens were included in the bioinformatics analyses per treatment for all analytics. Specific to beta-diversity, the qualitative metrics Bray-Curtis Dissimilarity Index (Fig. 2a) and Weighted Unifrac Distance Matrix (Fig. 2b) were statistically (*P* < 0.05) significant for the interaction of treatment and location. The statistical outputs for Bray-Curtis and weighted unifrac distance matrix are shown in Tables 1 and 2, respectively. There is a clear difference in beta diversity for both matrices between the ileum and jejunum (Table 1; Q < 0.05). Additionally, there are significant changes to diversity between the tissue within treatment (Table 2; Q < 0.05). Specific to the comparison between the 300 and 500 g/MT treatments, the 500 g/MT treatment was statistically significant between the ileum and jejunum (Q = 0.024). The 300 g/MT treatment also exhibited this difference. Likely, the effects of the local microbiota drive these differences, as indicated by the alpha diversity analysis.

**Table 1:** Bray-Curtis dissimilarity index statistical output

Group 1	Group 2	Sample Size	Permu-tations	R value	*p*-value	q-value*
**0 Ileum**	**0 Jejunum**	**10**	**999**	**0.64368999**	**0.001**	**0.00166667**
0 Ileum	300 Ileum	10	999	0.07736626	0.117	0.135
**0 Ileum**	**300 Jejunum**	**10**	**999**	**0.73744856**	**0.001**	**0.00166667**
0 Ileum	500 Ileum	10	999	0.01646091	0.356	0.38142857
**0 Ileum**	**500 Jejunum**	**10**	**999**	**0.61783265**	**0.001**	**0.00166667**
**0 Jejunum**	**300 Ileum**	**10**	**999**	**0.38518519**	**0.001**	**0.00166667**
0 Jejunum	300 Jejunum	10	999	−0.0292181	0.622	0.622
**0 Jejunum**	**500 Ileum**	**10**	**999**	**0.67530864**	**0.001**	**0.00166667**
**0 Jejunum**	**500 Jejunum**	**10**	**999**	**0.19862826**	**0.014**	**0.01909091**
**300 Ileum**	**300 Jejunum**	**10**	**999**	**0.45311111**	**0.001**	**0.00166667**
**300 Ileum**	**500 Ileum**	**10**	**999**	**0.13933333**	**0.024**	**0.03**
**300 Ileum**	**500 Jejunum**	**10**	**999**	**0.50977778**	**0.001**	**0.00166667**
**300 Jejunum**	**500 Ileum**	**10**	**999**	**0.74811111**	**0.001**	**0.00166667**
**300 Jejunum**	**500 Jejunum**	**10**	**999**	**0.24144444**	**0.006**	**0.009**
**500 Ileum**	**500 Jejunum**	**10**	**999**	**0.41222222**	**0.001**	**0.00166667**

*Bold indicates q < 0.05

**Table 2:** Weighted unifrac distance matrix statistical output

Group 1	Group 2	Sample Size	Permu-tations	R value	*p*-value	q-value*
**0 Ileum**	**0 Jejunum**	**10**	**999**	**0.4478738**	**0.002**	**0.006**
0 Ileum	300 Ileum	10	999	0.0436214	0.212	0.24692308
**0 Ileum**	**300 Jejunum**	**10**	**999**	**0.58573388**	**0.002**	**0.006**
0 Ileum	500 Ileum	10	999	−0.0633745	0.875	0.875
0 Ileum	500 Jejunum	10	999	0.24801097	0.017	0.02833333
**0 Jejunum**	**300 Ileum**	**10**	**999**	**0.32729767**	**0.003**	**0.0075**
0 Jejunum	300 Jejunum	10	999	−0.0403292	0.679	0.7275
**0 Jejunum**	**500 Ileum**	**10**	**999**	**0.45459534**	**0.001**	**0.005**
0 Jejunum	500 Jejunum	10	999	0.04389575	0.205	0.24692308
**300 Ileum**	**300 Jejunum**	**10**	**999**	**0.45022222**	**0.001**	**0.005**
300 Ileum	500 Ileum	10	999	0.04577778	0.214	0.24692308
**300 Ileum**	**500 Jejunum**	**10**	**999**	**0.23622222**	**0.008**	**0.01714286**
**300 Jejunum**	**500 Ileum**	**10**	**999**	**0.624**	**0.001**	**0.005**
300 Jejunum	500 Jejunum	10	999	0.11466667	0.075	0.1125
**500 Ileum**	**500 Jejunum**	**10**	**999**	**0.22244444**	**0.013**	**0.024375**

*Bold indicates q < 0.05

### Analysis of communities of the microbiota (ANCOM)

**Fig. 3:**
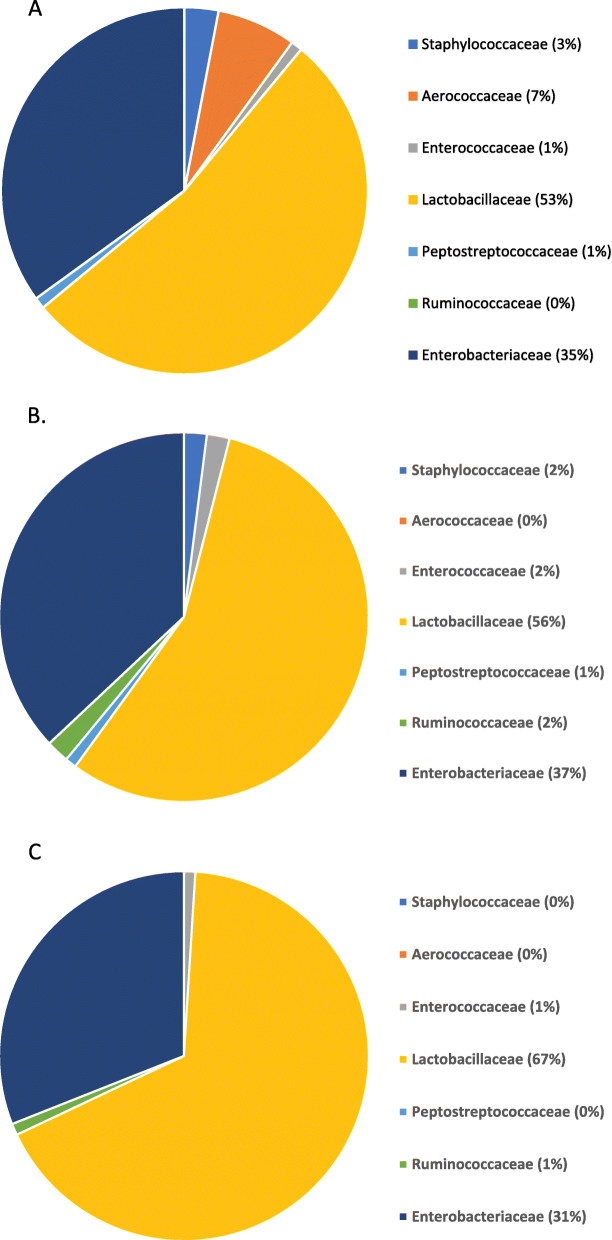
Analysis of communities of the microbiota (ANCOM) for jejunum samples. **a** NTC; **b** 300 g/MT; **c** 500 g/MT. The legends for the specific operational taxonomic units (OUT) associated with treatment as defined by ANCOM (Q < 0.05) is listed on the figure. Significant fluctuations occurred with increasing inclusion of the microencapsulated blend of organic acids and botanicals to the broiler diet

For each study (*n* = 2), a total of 5 samples were collected. In total, samples from 10 chickens were included in the bioinformatics analyses per treatment for all analytics. Because of the qualitative differences in beta diversity, and how there could be tissue-specific effects driving these differences, it became necessary to sort the data using ANCOM (Analysis of Communities of the Microbiota) to delineate the potential changes to compositional diversity. In the ileum, there was no difference in treatment by organ. Therefore, the differences in treatment observed in the beta diversity index are likely due to tissue-specific effects, not the localized effect of treatments. However, there were dose-dependent responses observed in the jejunum at the family level (Fig. 3). *Lactobacilliaceae* predominated for all three treatment groups, with a stepwise increase in this population from the NTC to the 300 g/MT and finally to the 500 g/MT group (Fig. 3a, b, c, respectively). This corresponds with a stepwise decrease in *Staphylococcaceae* from 3% in the NTC, 2% in the 300 g/MT and 0% (rounded number) of the total operational taxonomic units (OTU) associated with treatment at 500 g/MT. In relation to NTC (35%), the *Enterobacteriaceae* populations decreased in the 500 g/MT (31%) while the 300 g/MT increased (37%). In both the 300 g/MT and 500 g/MT treatments, the OTU identified as *Aerococcaceae* did not fluctuate (0%) compared to 7% in the NTC treatment. In relation to the three dietary treatments, *Ruminococcaceae* were more abundant in the 300 g/MT (2%) treatment compared to the samples collected from the NTC (0%) and 500 g/MT (1%) treatments. Therefore, there were significant (*Q* < 0.05) changes in the microbial consortia statistically associated with the treatment groups in the jejunum. These effects did not occur in the ileum, which suggests that the substantial difference in microbial consortia between tissues likely drives the beta diversity effects observed for the ileum.

### Linear discriminant analysis effect size (LEfSE) analysis

**Fig. 4:**
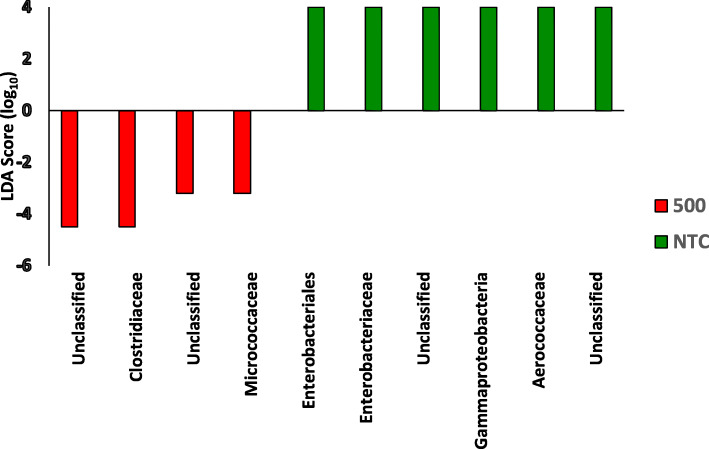
Linear discriminant analysis effect size (LEfSE) analysis. Missing operational taxonomic units (OUT) are not defined within the Family taxonomical designation and are labeled as unclassified. The 500 and NTC group are relative to 300. An LDA > +/− 2 with a Q < 0.05 is considered significant and is graphically represented. A negative linear discriminant analysis (LDA) score indicates a relative rise in population whereas a positive LDA score means the opposite. All comparisons are relative to 300 g/MT, which was selected as it is the intermediary dose and describes the potential dose effect

**Fig. 5:**
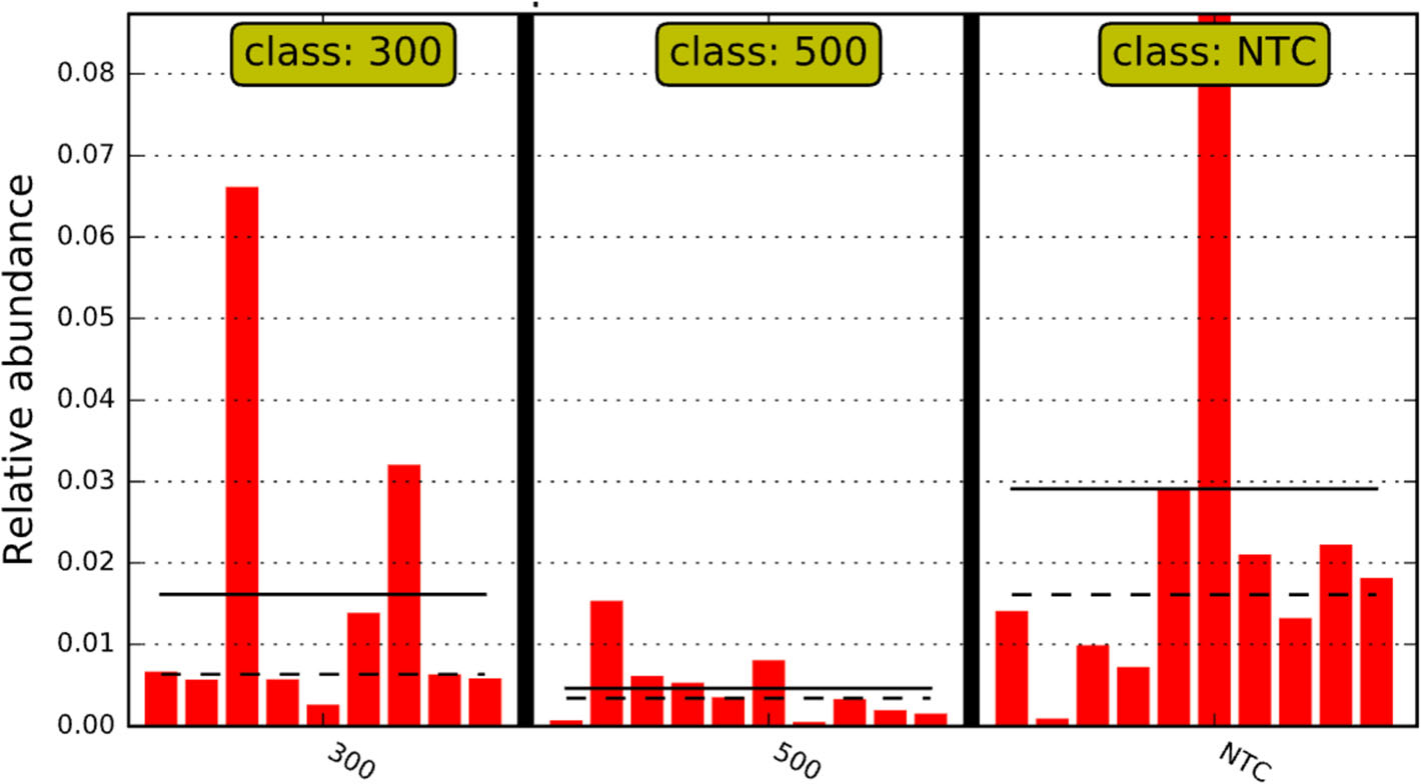
*Enterobacteriaceae* linear discriminant analysis effect size (LEfSE) relative abundance. The dotted lines are the median and the solid lines are the class mean. The relative abundance significant by LEfSE of each animal is displayed. The NTC has on average a greater abundance of *Enterobacteriaceae*, with a stepwise decrease in this population with increasing inclusion of the microencapsulated blend of organic acids and botanicals to the broiler diet

**Fig. 6:**
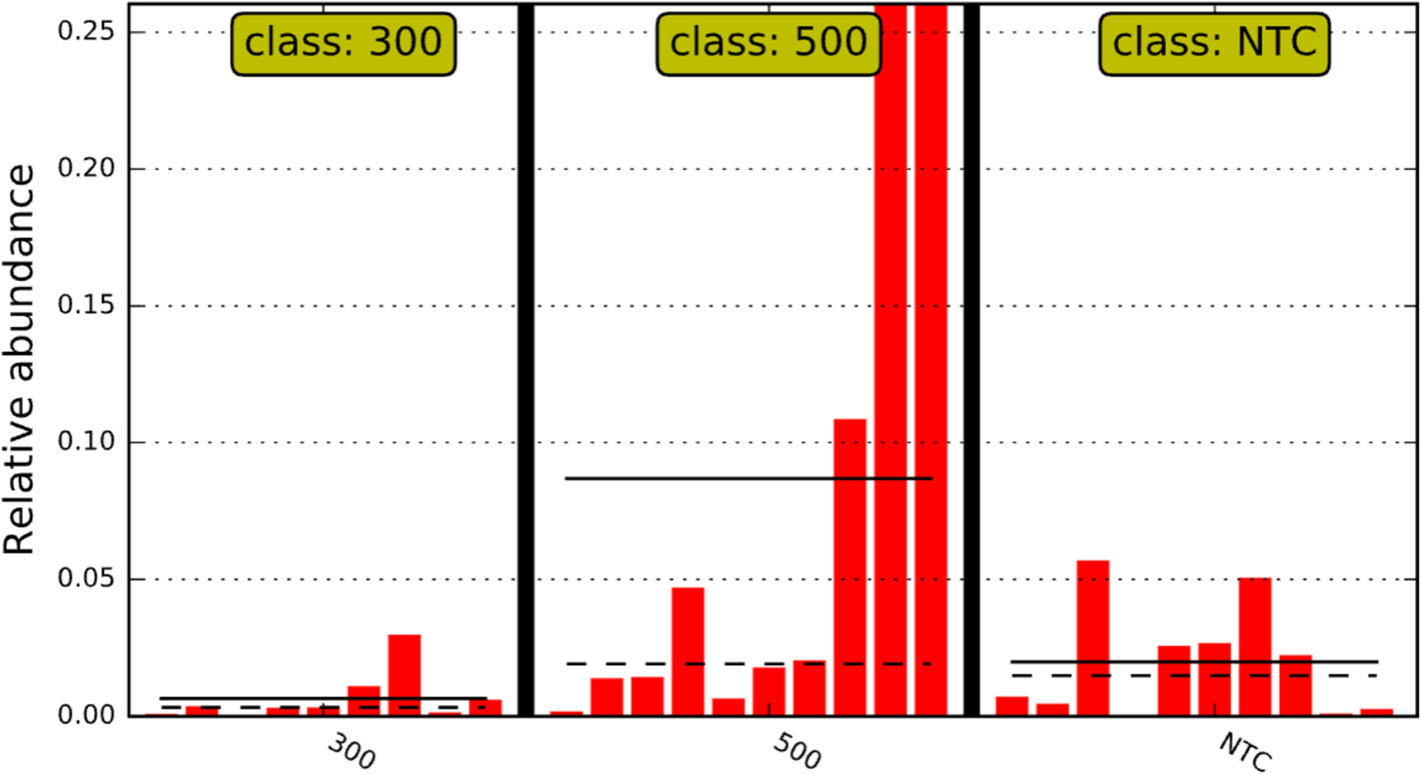
*Clostridiaceae* linear discriminant analysis effect size (LEfSE) relative abundance. The dotted lines are the median and the solid lines are the class mean. The relative abundance significant by LEfSE of each animal is displayed. The 300 g/MT treatment has on average a lower abundance of *Clostridiaceae*, with an increase at the 500 g/MT level

For each study (*n* = 2), a total of 5 samples were collected. In total, samples from 10 chickens were included in the bioinformatics analyses per treatment for all analytics. Least discriminant analysis effect size (LEfSE) accounts for underlying grouping by population; therefore, LEfSE indicates changes directly corresponding to treatment effects. As the 300 g/MT treatment was the intermediary treatment, the NTC and 500 g/MT scores were compared back to 300 g/MT (Fig. 4). The linear discriminant analysis (LDA) score relative to a certain treatment has an inverse relationship with relative abundance. The NTC exhibited an increase in the LDA of *Aerococcaceae*, *Gammaproteobacteria* and *Enterobacteriaceae* populations relative to the 300 g/MT group, which corresponds to a decrease in relative abundance (Fig. 4). Meanwhile, relative to 300 g/MT, the 500 g/MT treatment group had a lower LDA in *Clostridiaceae* and *Microccoaceae* which translates to an increase in relative abundance (Fig. 4). When parsing out important veterinary pathogens, *Enterobacteriacea*e demonstrated a stepwise decrease in relative abundance (from NTC onward) (Fig. 5). However, for *Clostridiaceae*, there was a significant increase in that relative population for 500 g/MT compared to the NTC and 300 g/MT treatments (Fig. 6), which is also supported by Fig. 4.

## Discussion

There are numerous studies by our laboratory and others highlighting the benefit of using encapsulated ingredients for targeted release in the poultry GIT [10, 21, 27, 30–35]. In a previous study, the blend of organic acids and botanicals evaluated herein enhanced gut immune and barrier function in the ileum and jejunum of weaned pigs [33]. In a separate study designed to begin to understand the mode-of-action at the gut level, a kinome analysis of ileal and jejunal samples collected from chickens revealed both common and distinct signaling pathways and proteins that were activated in each tissue segment compared to control-fed chickens [30]. Specifically, the Rap1 signaling pathway was activated compared to ileal samples [30] which could contribute to intestinal homeostasis and enhanced barrier function [36]. However, neither of the above-mentioned studies considered the role and impact on the gut microbial ecology; therefore, in the present study we determined the microbial populations of the ileum and jejunum from supplement-fed chickens compared to controls to provide additional insight.

The microencapsulated blend of organic acids and botanicals used herein is recognized by the European Union Commission and European Food Safety Authority ([EFSA]; AviPlus®P EFSA identification number 4d3) for its ability to enhance growth and feed efficiency in healthy chickens. In the current study, there were no weight differences (*P* > 0.05) between the supplement- and control-fed chicks, but the chicks on the supplemented diets were slightly heavier than the NTC in a dose-dependent manner. The fact that there were no statistical differences in weight is not surprising since the study was terminated at 15 days which is similar to another feed additive study carried out for 14 days [37]. Broiler studies focused on growth and performance are typically carried out to 35–42 days with incremental measures incorporated into the study design [10, 28, 38]; however our findings do suggest early shifts in the jejunal microbial populations and signaling pathways [30] could be contributing to the enhanced growth and performance that was observed during the EFSA approval process. In fact, in a study conducted on broilers fed with incremental doses of the same microencapsulated blend of organic acids and botanicals demonstrated a linear increase of body weight and improvement of feed efficiency starting at 7 days and reaching the maximum effect at 35 days [29]. The performance results were correlated to an increase in *Lactobacillus* counts in the feces thus suggesting the change in microbial populations as one of the factors contributing to the overall result at the completion of the grow-out. Nevertheless, microbial populations are just one of the factors determining growth performance as there are also important contributions coming from the host, the environment, and the complex group of interactions among these elements. As one study cannot unveil and explain the complexity of all of these interactions, with the present study we wanted to understand the impact of microencapsulated organic acids and botanicals on the jejunum and ileum populations at the early stages of development of broiler chickens irrespective of growth performance at the end of grow-out. The limitation of this kind of approach resided in the lack of correlation between the changes we observed at day 15 days and the impact these might have had on the final performance. To more definitively address the correlation between microbial populations and performance, time course studies with additional biological replicates and increased numbers of chicks need to be conducted through the entire grow-out. Poultry feed additive studies typically focus on one or more aspect of performance (body weight or feed efficiency); however, the objective of the current project sought to follow-up on the earlier kinome study where signaling differences were observed at the tissue level (ileum versus jejunum) which, as we have shown herein, tissue-specific differences were also observed in the microbial populations.

Evidence suggests the stability of the microbiota is defined over time; however, changes observed in stable systems by compartment (tissue segment) can also indirectly support what is or is not viewed as a stable microbiota that may contribute to a loss in homeostasis [39, 40]. A classic example of the breakdown of gastrointestinal homeostasis is the emergence of ecological dysbiosis resulting in the de-compartmentalization of the gastrointestinal microbiota [19, 41]. The current study was not focused specifically on homeostasis or dysbiosis over time, but the findings herein indicate dietary supplementation with organic acids and natural compounds did result in significant compartmentalization of the microbial ecology within the ileum and jejunum of chickens. Each compartment functions independently with nutrient digestion and absorption typically occurring in the jejunum with water and mineral adsorption generally taking place in the ileum [42, 43]; therefore, it would be expected that the microbial populations would, in fact, differ between the two compartments. Additionally, while tissue differences in the microbial makeup exist comparing the NTC to tissue from supplement-fed chickens, there is not a collapse and shrinkage in diversity or a bloom of populations. These data are in agreement with other studies looking into nutrition and gastrointestinal health studies [39, 40]. While we cannot speak to potential changes or stability over time, data presented does indicate the microbiota is biologically diverse at 15 d-of-age in chicks provided a diet supplemented with the microencapsulated blend of organic acids and botanicals. However, some authors suggest the gut and microbiota at 15 d-of-age is only semi-developed [44]; therefore, future studies should consider the microbiota populations over the typical 42-day grow-out period.

Alpha diversity speaks to the community structure and evenness of the microbial ecosystem without taking into account differences in speciation while Shannon’s diversity index is classically associated with numerous microbial studies and is used to calculate evenness [16]. Beta diversity indicates there may be compositional differences that are arising, with Bray-Curtis being a function of total assessment and the Weighted Unifrac Distance Matrix considering phylogenetic branch length and both are considered qualitative as total reads and counts leading to the differences are not considered [16]. Dietary supplementation with natural compounds including organic acids and essential oils does not always result in changes to alpha and beta diversity in microbial populations within the poultry GIT [10]. However, the blend of organic acids and botanicals used in the current study, produced an increase in diversity and evenness for the jejunum compared to the ileum. Similarly, in other pharmacological studies, the biotransformation of drugs by the microbiota results in their absorption in the jejunum and are linked to increased diversity and biological activity of the microbial population [45, 46]. The jejunum is the main sight for nutrient absorption in poultry [42], as well as in mammals, and it has been suggested that the jejunum is the most logical site to observe treatment effects [13] which is what we observed in the current study. Also, some feed additive studies utilize traditional culture-dependent microbiological evaluation to characterize the GIT microbial populations [28, 38, 47]. While these studies are valid and valuable, they are unable to take into account compositional and diversity changes. Therefore, the culture-independent study herein provides a deeper insight into the complete microbial shifts in two diverse and bioactive components of the GIT.

Natural compounds such as oregano and its derivatives, including thymol and carvacrol, are recognized for their potential benefits to the poultry industry because of antimicrobial properties and animal health benefits [9]. Additionally, dietary supplementation with thymol has shown to increase *Lactobacillus* populations in the ileum [24, 47]; however, in the current study the changes in *Lactobacillus* populations were more pronounced in the jejunum compared to the ileum. This dissimilarity is likely attributed to experimental design differences including, but not limited to, the delivery method (non-encapsulated vs encapsulated), the genetic line of chickens used (Arbor Acre vs Cobb), the feed additive, or the thymol concentration (25% vs 1.7%). Even though the tissue-specific changes were different than the aforementioned study, our findings are in agreement with another study showing that inclusion of thymol does alter the GIT microflora of poultry [23]. Another natural compound, a green tea component, also resulted in increased *Lactobacillus* in the jejunum compared to the ileum when fed to chickens [48]. Collectively, these studies indicate an important role for the inclusion of thymol and other natural compounds into the diet as antibiotic alternatives.

In addition to increased *Lactobacillus* populations, other favorable changes were observed following supplementation including significant changes in Clostridiaceae in the 500 g/MT jejunal samples. Similarly, supplementation with eugenol, an essential oil, increased members of the Clostridiales order in mice that proved protective against pathogenic challenge [49]. There are a number of studies employing supplementation with natural products including organic acids and phytochemicals that show improvements to intestinal integrity as well as protecting against the pathology and loss of performance associated with necrotic enteritis in broilers [10, 24, 35, 50]. Future challenge trials will be conducted to determine if the blend of organic acids and botanicals used herein confers protective effects against *Clostridium perfringens*-induced necrotic enteritis and what role the GIT microbial populations play in determining disease outcome. Ruminococcaceae families (300 and 500 g/MT) also increased in our study that was accompanied by a decrease in Enterobacteriaceae (in the 500 g/MT dose). These data are in agreement with recent studies that also fed diets that incorporated an encapsulated blend of organic acids and essential oils [10] and phytonutrients [49]. The organic acids and essential oils were different, but the beneficial effects were similar which is also supported by numerous studies using diverse organic acids including, but not limited to, butyric acid [38], encapsulated benzoic acid [27], or formic and propionic acids [28] to enhance the GIT microbiota, poultry health, and performance. Collectively, the data presented herein, along with supporting studies in the literature, demonstrate the importance of targeted release of natural compounds in the poultry GIT to maximize efficacy and potential benefits to the bird. It has been said “increased understanding of how the microbiota interacts with animal hosts will improve microbiome intervention strategies to mitigate production losses” [51]. This statement becomes even more critical as antibiotic use is further curtailed and restricted within the poultry industry, and the present study begins to understand the host-microbiome interaction in the presence of natural antibiotic alternatives.

As with any laboratory-controlled experiment, there are limitations that prohibit the inclusion of all variables encountered on the farm under commercial conditions. One of the most obvious discrepancies would be the environment where the newly hatched chick is placed. Under commercial conditions chicks would be placed in a house that has been exposed to thousands of chickens compared to an experimental room that is thoroughly disinfected prior to placement of chicks onto clean litter versus some commercial settings where chicks would be placed on used litter. Clearly, these differences would likely impact the outcome of a microbiome experiment due to the immediate exposure to the myriad of microorganisms found in a poultry house; however, this does not diminish our findings as clear compartmentalization of the microbial populations between the ileum and jejunum were observed. One approach to mitigate this experimental limitation would be to place the chicks on used litter to more realistically mimic the early GIT colonization seen under commercial conditions; however, this approach introduces uncontrollable variables making reproducibility of results difficult. With respect to the microbiome analysis approach that was employed, one of the limitations is the results are qualitative which does not take into consideration cell counts and 16S ribosomal DNA (rDNA) copy number. Different populations can contribute varying copy numbers of 16S rDNA to the analysis; therefore, using quantitative methods will become more important and commonplace as microbiome studies evolve and technologies advance [52]. Additionally, the ability to utilize long read technology will also become necessary to truly understand microbial shifts due to treatment, instead of sequencing small variable regions, such as V3 or V4; however, the approaches we employed are widely accepted and are common practice today [48, 53]. Despite the above-mentioned limitations, the observed changes in beta diversity will remain consistent and are indicative of potentially optimal microbiota changes. Further, this study demonstrated that shifts in dispersion and mean, as analyzed by ANISOM, occurred by treatment. This type of metric will also stand the test of time and prove essential in delineating the biological role of the microbiota and how it is affected by treatment. It should also be noted that these limitations exist with all currently conducted microbiota studies that are not commercially derived, with many of these limitations existing for decades. Yet, studies such as the one conducted here are considered academically sound and important for the industry. While microbiota studies will become more advanced as technology and bioinformatics improves, the importance of academically derived studies independent of field conditions will always be important and relevant.

Future studies considering the impact of the biochemical and/or metabolites produced in each compartment of the GIT would provide additional mechanistic insight. Studies in the literature show changes to the microbial populations could diffuse outward or that the metabolites are further transformed by downstream microbial populations impacting colonization by foodborne pathogens such as *Salmonella* [54, 55]. Dietary supplementation with organic acids and botanicals significantly lowers *Salmonella* [31] and *Campylobacter* [32] colonization in market-age broilers. Though not considered in those earlier studies, it is possible that changes to the GIT microbial populations while the bird is developing could have contributed to the observed decreases in *Salmonella* and *Campylobacter* colonization, but additional studies are required to confirm this hypothesis. Studies support there is compartmental activation of the microbiota; but ultimately it will be the resulting physiological effects within the different compartments as they carry out their specific biological processes [46, 56] that will have the greatest impact.

Finally, although outside the scope of the current manuscript, the authors recognize the importance of determining feed efficiency, nutrient absorption, and other GIT functionality traits as predictors and contributors to the final growth performance and on-farm profitability.

## Conclusions

The bioactive site for the microencapsulated blend of organic acids and botanicals used in this study is in the jejunum, which is also the site of nutrient absorption. Understanding these fundamental changes to the microbiota composition of the ileum and jejunum indicate future studies should consider evaluating the metabolome which will provide a deeper understanding of the impact of organic acids and botanicals. However, based on the changes shown herein, the data indicate inclusion of the microencapsulated blend of organic acids and botanicals does enhance the GIT microbiota and may be a viable antibiotic alternative for use in the poultry industry.

## Methods

### Experimental design, animal husbandry, and tissue collection

The experiments were conducted in accordance with the recommended code of practice for the care and handling of poultry and followed the ethical principles according to the *Guide for the Care and Use of Laboratory Animals* [57]. All bird studies were under the approved experimental procedures outlined in protocol #2017008 and were approved by the USDA/ARS Institutional Animal Care and Use Committee and overseen by Dr. Roger B. Harvey, DVM (attending veterinarian).

Day-of-hatch by-product male breeder chicks were obtained from a commercial hatchery (Timpson, TX, USA), and were not vaccinated at any point during the study. The chicks were transported in standard chick boxes and placed in a BL2 building in floor pens (3 m × 3 m) containing wood shavings and provided supplemental heat and ad libitum access to food supplied in hanging feeders and fresh water through nipple drinkers. Chickens were provided 24 h of continual light at placement to ensure sufficient water and food intake, then transitioned to 18 h of light and 6 h of darkness for the remainder of the study. The temperature of the pens was maintained at 35 °C for day 1 to 3, 32 to 34 °C for day 4 to 7, and 29 to 31 °C for day 8 to 15. Chickens were monitored each morning (08:00) for mortality, behavioral changes, litter quality, and feed and waterers were checked to ensure they were in proper working order. No mortality, behavioral changes, or other animal welfare concerns were observed during the study. The chicks were not treated with any medications or other therapeutic interventions during the study. No antibiotics were given to the chicks nor included in any of the diets used in the study.

Two independent trials were conducted using chicks from a different hatch-out. The chicks were weighed at placement (day of hatch) and at the conclusion of the study (d15). Chickens from the two hatches were maintained separately to ensure proper biological replication of the experiment. The two replicates of the experiment were handled as follows: chickens (*n* = 15) were randomly selected and placed into one of three groups: the NTC (0 g/MT AviPlus®P; *n* = 5 chickens) or one of the experimental groups (300 g/MT; *n* = 5 chickens; 500 g/MT AviPlus®P; *n* = 5 chickens). The experiment was conducted using two replicate pens therefore 10 chickens/treatment were used for all analyses. Chickens assigned to the control pen were allowed ad libitum access to a balanced, un-medicated, antibiotic-free corn and soybean meal-based starter diet that met or exceeded the established nutrient requirements [58]. Chickens assigned to the supplement-fed pens were given free access to the same starter diet mixed with 300 or 500 g/metric ton (MT) of a microencapsulated blend of citric (25%) and sorbic (16.7%) acids, thymol (1.7%), and vanillin (1.0%) (AviPlus®P, Vetagro S.p.A., Reggio Emilia, Italy). The remaining 55.6% of the feed additive is comprised of hydrogenated vegetable fats. The feed was mixed in small batches for 15 min (34 g AviPlus®P/113 kg feed and 56.7 g AviPlus®P/113 kg feed for the 300 and 500 g/MT, respectively) using a Wenger AB batch mixer (Sebetha, KS). The control diet was mixed first to ensure consistency of the mash supplied to each group of chicks.

All chickens assigned to the control pens were evaluated first followed by those in the 300 and 500 g/MT groups. For both experimental replicates, chickens on the control, 300, and 500 g/MT diet (*n* = 5 per group/experiment; *n* = 10 total) were euthanized by cervical dislocation and necropsied at 15-days-of-age. The ileum and jejunum were selected because they are two important organ systems associated with feed efficiency and production in broilers. In relation to Meckel’s diverticulum, the jejunum sample was collected approximately 10 cm proximal and the ileum sample was collected approximately 10 cm distally. Total content from these regions of the jejunum and ileum were collected and immediately flash frozen in liquid nitrogen to preserve activity followed by transfer to − 80 °C until further processing and analysis. Samples were collected at day 15 based on previous work [33, 34] and in consideration of the productive cycle of broilers. In commercial settings, most diet changes going from the starter to grower occurs between 10 and 15 days-of-age. The first two weeks are very critical to the development of the gastrointestinal and immunological function and by 2 to 3-wk-of-age broilers have a diversified microflora.

### DNA extraction

The DNA was extracted and sequenced as per standard laboratory guidelines [59]. Briefly, the tissue (ileum or jejunum) contents were thawed, homogenized, and 0.3 g removed followed by extraction using the Qiagen Stool Kit (Qiagen, Hilden, Germany). The DNA was eluted and stored at − 20 °C until the library preparations commenced. Using the amplicon sequence variance index primers and protocol, the library was prepared as previously described [60]. Normalization and library clean-up were also performed prior to sequencing [59, 60]. The Illumina MiSeq 16S rDNA Microbiome Library (version 2; Illumina, San Diego, CA, USA) was constructed and sequenced as per standard company guidelines. The sequences were exported from Illumina BaseSpace [61], de-multiplexed, and prepared for import into QIIME2.2019.1 (quantitative insights into microbial ecology) [62].

### Microbiome analyses

Each bird sample was handled on an individual basis (*n* = 10) and each tissue (ileum and jejunum) was kept separate for all analyses. The sequences were filtered for quality and chimera using divisive amplicon denoising algorithm (DADA2), with Q30 being the cut off range for sequence quality [63]. Additionally, in order to remove any potential chimeras that escaped detection, OTUs with a frequency of less than 3 were removed from the analyses. Alpha and beta analyses were performed using the standard QIIME2.2019.1 pipeline, with ANOSIM (analysis of similarities) selected as it considers dispersion and the mean difference in beta diversity per group. To refine the analyses, the tissue data was then sorted into either a “ileum” or “jejunum” dataset for compositional analysis. Differential abundance was evaluated using the plugin ANCOM [64], which considers the compositional changes associated with treatment. Finally, LEfSE analysis was performed per standard practices [65] to determine which populations were enriched by treatment using LDA, which is inversely related to ANCOM data [66].

### Statistical analyses

Compositional microbiota studies are necessarily heterogeneous and represent the changes of a microbial consortia and structure by treatment. Therefore, the use of statistically sound plugins to evaluate the compositional data are important as standard statistical practices are irrelevant if they do not take into account the compositional nature of the data. Alpha and beta diversity parameters were considered significant if the main effect was *P* < 0.05. Pairwise differences between the main effect of treatment were considered significant if Q < 0.05, which takes into account the false discovery rate associated with this class of data. The Kruskall-Wallis test was used in the alpha diversity metrics, meanwhile the ANISOM test was used for the beta diversity tests as per the standard QIIME2.2019.1 pipeline. The Q-value is representative of the corrected *p*-value, which is a standard component of multivariate and multihypothesis-based testing associated with this kind of data set. Finally, for ANCOM, PROC GLM was used in the background, with the central log2 ratio of the effect (W) significant of Q < 0.05 evaluating the changes in the microbial consortia by treatment. Therefore, any OTUs arising from the analyses fluctuate statistically by treatment and are not quantitative differences between each treatment as the entirety of the microbial consortia fluctuation by treatment is what is regarded as significant in this analysis (*Q* < 0.05). Pairwise differences between the treatment groups was instead performed by LEfSE, which is an independent analysis but provides species differences, which was considered significant if the LDA > +/− 2 and *Q* < 0.05 at the family level. Initially, the analysis was relative to the 300 g/MT group and important microbial families associated with poultry production were identified for further analyses. It should also be noted that a negative LDA score relative to a comparison indicates an increase in relative abundance, with a positive score meaning the opposite.

## Data Availability

Data is freely available at www.github.com/RickeLab/BMCMicroSubmission26Feb2020. Please contact SCR if you request additional information.

## Abbreviations

ANCOM: Analysis of communities of the microbiota
ANOSIM: Analysis of similarities
DADA2: Divisive amplicon denoising algorithm
EFSA: European Food Safety Authority
GIT: Gastrointestinal tract
LDA: Linear discriminant analysis
LEfSe: Linear discriminant analysis effect size
NTC: No treatment control
OTU: Operational taxonomic unit
QIIME: Quantitative insights into microbial ecology
rDNA: Ribosomal DNA
